# Transgene Regulation Using the Tetracycline-Inducible TetR-KRAB System after AAV-Mediated Gene Transfer in Rodents and Nonhuman Primates

**DOI:** 10.1371/journal.pone.0102538

**Published:** 2014-09-23

**Authors:** Caroline Le Guiner, Knut Stieger, Alice Toromanoff, Mickaël Guilbaud, Alexandra Mendes-Madeira, Marie Devaux, Lydie Guigand, Yan Cherel, Philippe Moullier, Fabienne Rolling, Oumeya Adjali

**Affiliations:** 1 INSERM UMR 1089, Atlantic Gene Therapies, Nantes University Hospital, Nantes, France; 2 Department of Ophthalmology, Faculty of Medicine, Justus-Liebig-University Giessen, Giessen, Germany; 3 INRA UMR 703 and Atlantic Gene Therapies, ONIRIS, Nantes, France; 4 Department of Molecular Genetics and Microbiology department, University of Florida, Gainesville, Florida, United States of America; University of Florida, United States of America

## Abstract

Numerous studies have demonstrated the efficacy of the Adeno-Associated Virus (AAV)-based gene delivery platform *in vivo*. The control of transgene expression in many protocols is highly desirable for therapeutic applications and/or safety reasons. To date, the tetracycline and the rapamycin dependent regulatory systems have been the most widely evaluated. While the long-term regulation of the transgene has been obtained in rodent models, the translation of these studies to larger animals, especially to nonhuman primates (NHP), has often resulted in an immune response against the recombinant regulator protein involved in transgene expression regulation. These immune responses were dependent on the target tissue and vector delivery route. Here, using AAV vectors, we evaluated a doxycyclin-inducible system in rodents and macaques in which the TetR protein is fused to the human Krüppel associated box (KRAB) protein. We demonstrated long term gene regulation efficiency in rodents after subretinal and intramuscular administration of AAV5 and AAV1 vectors, respectively. However, as previously described for other chimeric transactivators, the TetR-KRAB-based system failed to achieve long term regulation in the macaque after intramuscular vector delivery because of the development of an immune response. Thus, immunity against the chimeric transactivator TetR-KRAB emerged as the primary limitation for the clinical translation of the system when targeting the skeletal muscle, as previously described for other regulatory proteins. New developments in the field of chimeric drug-sensitive transactivators with the potential to not trigger the host immune system are still needed.

## Introduction

Long-term expression of transgenes can be supported by recombinant Adeno-Associated Virus (rAAV)-derived vectors in a variety of target organs in mammals, including human patients, after a single administration [1], [2]. Vector genomes persist predominantly as episomal monomeric and concatemeric circles [3], and integrative events have been demonstrated to be rare in nonhuman primate (NHP) liver and muscle with no preference for specific genomic *loci* [4]. Recently, the first rAAV-based clinical product (Glybera) received market authorization for the treatment of lipoprotein lipase deficiency [5], [6]. The clinical safety profile and the application range of rAAV-based gene transfer strategies will be broadened by using regulatable systems, enabling the fine-tuning of therapeutic gene expression. In particular, the control of gene expression is crucial in situations where a specific therapeutic window is required to avoid transgene toxicity. However, a limited number of clinically translatable regulatory systems are available. All of these systems use chimeric transactivators, the activity of which is controlled by drugs, such as tetracycline and its derivative doxycycline (Dox) or rapamycin [7], [8], [9], [10], [11], [12] for the two most developed systems. Most applications have been in experimental studies to demonstrate the feasibility of this approach *in vitro* or *in vivo* in rodent models, but few have been translated into large animal models. Our laboratory, in a variety of experimental settings, has evaluated the Dox-regulatable Tet-ON system in rodents and NHP models using the rAAV platform. The general configuration of our vector construct consisted of a single expression cassette flanked by the AAV2 Inverted Terminal Repeat (ITR) sequences and containing the transgene of interest and the chimeric transactivator rtTA. The transgene of interest was placed under the control of the doxycycline-inducible P_tet-1_ promoter, in which Tet operator (*TetO*) sequence concatemers are fused to the minimal promoter sequence derived from the cytomegalovirus (CMV) immediate-early promoter. In the same vector, the chimeric transactivator rtTA was under the control of either a constitutive ubiquitous or a tissue-specific promoter, such as the desmin promoter, when the latter construct was meant to be more specifically functional in the skeletal muscle [13], [14], [15], [16]. It was demonstrated that when expressed from the retinal-pigmented epithelium (RPE) and the neuroretina [16], [17], [18], tight and permanent transgene regulation was consistently achieved in 100% of the experimental NHP, without eliciting an immune response against the transactivator. Conversely, when the expression cassette was expressed from the skeletal muscle after intramuscular (IM) delivery of the vector, the majority (85%) of the NHP mounted an immune response against the Dox-sensitive rtTA transactivator, resulting in the rapid loss of reporter transgene regulation despite Dox administration to the macaques [13], [14], [19]. The factors involved in this immunotoxicity are numerous, but the target organ and the route of administration are likely to play a critical role. The rtTA is a hybrid molecule obtained after fusion of the DNA binding domain of the repressor from the Tn10 tetracycline-resistance operon of *E. coli* (TetR) to the C-terminal portion of VP16 of herpes simplex virus (HSV). The rtTA epitope(s) - either originating from one of the two domains or both - recognized by the macaque immune system remains unknown, but if the transactivator domain of the molecule bears the dominant epitopes, then using a less antigenic transactivator protein would potentially be beneficial to support long-term transgene regulation.

By fusing the KRAB domain of the human zinc-finger protein Kox1 to the DNA binding domain of the Tet repressor, Deuschle *et al.* generated a Dox-sensitive transrepressor called TetR-KRAB [20]. Krüppel associated box (KRAB) is an approximately 75-amino acid transcriptional repression domain found in many mammalian zinc finger-containing proteins, which can suppress, in an orientation-independent manner, polymerase I, II and III-mediated transcription within a distance of up to 3 kb from its DNA binding site, presumably by triggering the formation of heterochromatin [20], [21], [22]. An understanding of the mechanism of action of KRAB has been achieved through the identification and characterization of KRAB-associated protein 1 (KAP1), believed to represent its universal corepressor [23]. In the KRAB-based regulation system, TetR-KRAB binds specifically to the *TetO* sequences in the absence of Dox, thus suppressing the activity of the nearby promoter(s). In contrast, upon Dox administration, TetR-KRAB is sequestered from *TetO* sequences, allowing transgene expression after KRAB-mediated transcription repression is lifted [20], [21], [22]. Using the lentiviral vector platform, the system allowed concise *in vitro* gene expression switch even when low amounts of Dox were added to the culture [24] and over several induction cycles [25] . The TetR-KRAB drug inducible system has also been demonstrated to be an efficient, versatile tool in rodent models in the context of lentiviral vector-based gene transfer [26]. Initially, it was believed that *TetO*-linked transcriptional units were repressed by TetR-KRAB only when integrated into the genome [24] in an independent integration site manner [27]. Since then, this has been refuted, as Barde *et al*. [28] demonstrated that TetR-KRAB can also regulate transgene expression *in vitro* and *in vivo* in a mouse model, using either integration-deficient lentiviral vectors or rAAV-based vectors. Recently, another study demonstrated the functionality of the TetR-KRAB repressor-based system after IM delivery of a rAAV2/8 vector in the mouse [29].

In this study, we evaluated the ability of the TetR-KRAB system to mediate concise and reproducible transgene transcriptional regulation after subretinal rAAV delivery in rats. Because the efficiency and immunogenicity of the TetR-KRAB based-system has not been explored in higher species, we also evaluated the system after IM delivery in mice *versus* a macaque model. Because the human and putative macaque Kox1 nucleotide sequences are more than 95% homologous, we hypothesized that exchanging the HSV VP16 domain with the human KRAB one may result in less immunotoxicity than when the rtTA transactivator is expressed from the macaque skeletal muscle.

## Results

### rAAV.TetR-KRAB/GFP in the rat retina results in long term Dox-mediated transgene regulation

**Figure 1:**
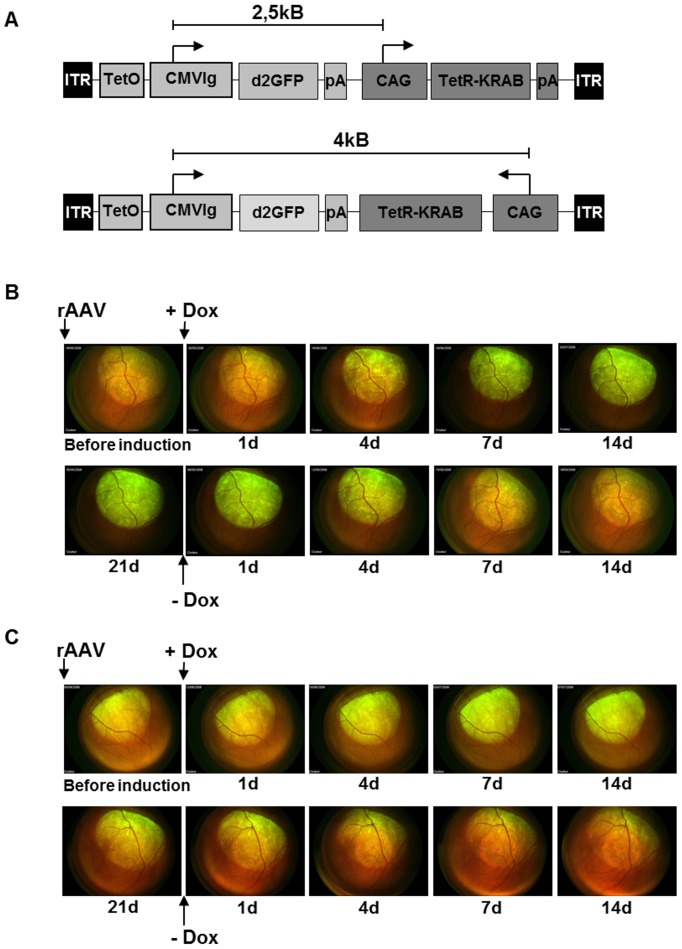
rAAV.TetR-KRAB/d2GFP in the rat retina results in Dox-mediated transgene regulation. (**A**) Vector design: Vectors encode the destabilized GFP (d2GFP) under the control of the *TetO*.CMVlg promoter and the trans-inhibitor TetR-KRAB under the control of the CAG promoter. pA (d2GFP), SV40 polyadenylation signal; pA (TetR-KRAB), bovine growth hormone (BGH) polyadenylation signal; pA (bidirectional), SV40 polyadenylation signal; ITR, inverted terminal repeat of AAV2. Both expression cassettes *TetO*.CMVlg-d2GFP-pA and CAG-TetR-KRAB-pA were cloned between AAV2 ITRs either in the same orientation (*forward*) using two pA signals or in opposite orientations (*opposite*) using a bidirectional pA. Arrows indicate the transcription start sites, and the distance between the two promoters is indicated above both constructions. (**B and C**) Fluorescence retinal images showing induction and de-induction kinetics of d2GFP expression in the retina of representative rats subretinally injected with (**B**) rAAV5.d2GFP.KRAB *forward* and (**C**) rAAV5.d2GFP.KRAB *opposite*. A volume of 2.5 µL of vector solution was injected subretinally in 12 rats (n = 6 for each vector). Dox at a dose of 10 mg/kg/day was added to (+Dox) or removed from (-Dox) the drinking water, as indicated by the arrows. d2GFP expression was monitored in representative rats by direct retinal imaging after vector delivery (before induction) and following Dox administration or withdrawal during 14 to 21 days at the indicated time points.

For retinal gene transfer, we used rAAV vectors in which the TetR-KRAB expression was driven by the ubiquitous CAG promoter and the *d2GFP* reporter gene was under the control of the *TetO*-cytomegalovirus (CMV) entire natural promoter (CMVlg). The destabilized GFP variant d2GFP has a reduced half-life (2 hours instead of >24 hours for the standard GFP [30]) and was used in order to improve the accurate analysis of gene expression kinetic. Two different plasmid constructs were generated containing these two expression cassettes, TetO.CMVlg-d2GFP-pA and CAG-TetR-KRAB-pA, either in the same (*forward*) or in the opposite (*opposite*) directions (**Figure 1A**). In the *forward* construct, the CAG promoter, driving TetR-KRAB expression, was located 2.5 kB from the *TetO* sequences. Because the TetR-KRAB protein has been demonstrated to be capable of inhibiting all promoters within at least 3 kB [20] and the *forward* construct would therefore inhibit the CAG promoter, we generated the *opposite* construct, where the two expression cassettes are cloned in a manner where both promoters are at the opposite ends at a distance of 4 kB. Using these two expression cassettes, we evaluated two different rAAV5 vectors: rAAV5.d2GFP.KRAB *forward* and rAAV5.d2GFP.KRAB *opposite*. Serotype 5 was used due to its tropism for the retina with an efficient transduction in both the retinal-pigmented epithelium cells and the photoreceptors [31]. Both vectors were injected subretinally into two groups of 6 Wistar rats, and the retinas were routinely analyzed by direct *in vivo* fluorescence imaging to directly monitor the appearance of GFP expression. A weak GFP signal, representing the background levels of protein expression in the absence of induction, appeared in all retinas within 2 months following vector injection (**Figure 1B and 1C** **, before induction**) compared with non-injected rats **(data not shown).**

Upon induction with 10 mg/kg/day of Dox by oral administration *via* the drinking water, the GFP signal in the retina increased and reached a maximum within 14 days (**Figure 1B and 1C** **, + Dox**). The signal remained stable during the induction period of 3 weeks. Within 14 days after Dox withdrawal, the GFP signal decreased progressively to the baseline levels observed before induction (**Figure 1B and 1C** **, -Dox**). The kinetics of the regulation of transgene expression was similar for both groups of rats, either injected with the *forward* (**Figure 1B**) or *opposite* (**Figure 1C**) constructs. The quantification of GFP fluorescence further confirmed these findings (**Figure S1A**).

**Figure 2:**
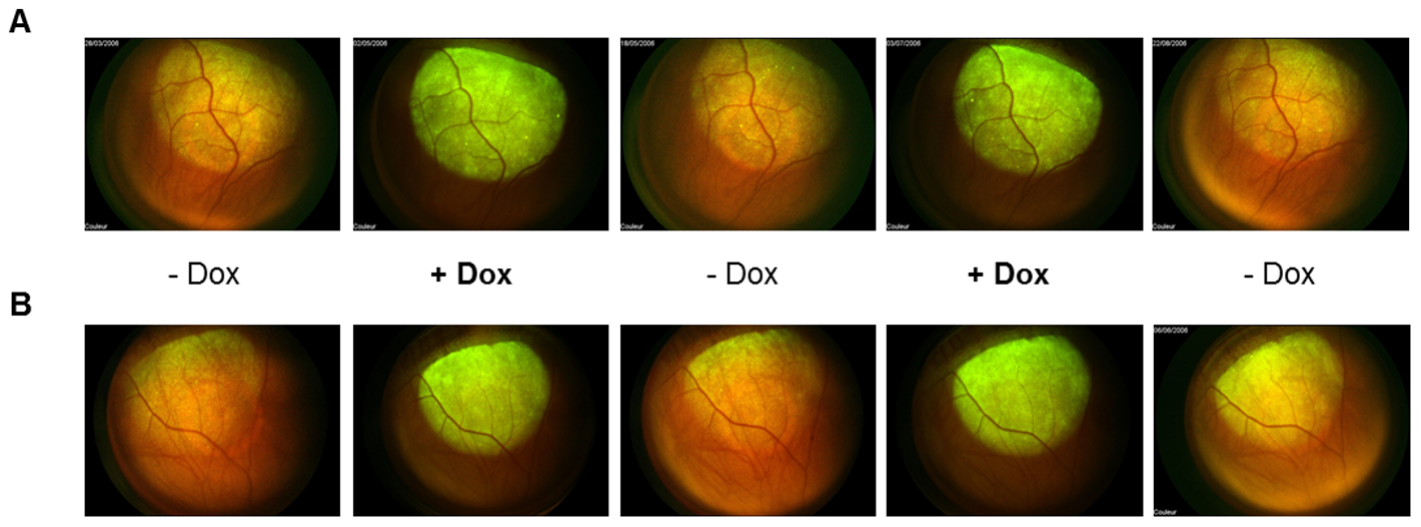
Regulation of d2GFP expression in the rat retina using the TetR-KRAB system over a time period of 48 weeks. Expression of d2GFP was induced several times with Dox for over 48 weeks to evaluate the long-term regulation and functionality of the regulatory system after subretinal injection of (**A**) rAAV5.d2GFP.KRAB *forward* and (**B**) rAAV5.d2GFP.KRAB *opposite* vectors. Images of fundus fluorescence were taken 14 days after starting the administration of Dox (+Dox) or the withdrawal of Dox (-Dox) at 15, 20 and 38 weeks post-injection.

All rats, except one control rat for each group, were subjected to repeated Dox exposures over a one-year period, and retinas were analyzed by *in vivo* retinal imaging to monitor GFP expression kinetics (**Figure 2**). As expected, the non-induced control rat did not show fluorescence variation during this long period **(data not shown)**. In contrast and similarly to the first induction, GFP expression increased upon Dox administration in the other rats within 14 days to a maximum, remained stable over 3 weeks (duration of induction) and decreased within 14 days upon Dox withdrawal. This persisting gene regulation was further confirmed by the quantification of GFP fluorescence, with a slighltly more stringent regulation achieved with the *forward* vector (**Figure S1B**). Thus, TetR-KRAB responsiveness to doxycycline was maintained over time for up to one year for the *forward* and *opposite* vectors.

### AAV.TetR-KRAB/Epo in murine muscle results in long-term Dox-mediated transgene regulation

**Figure 3:**
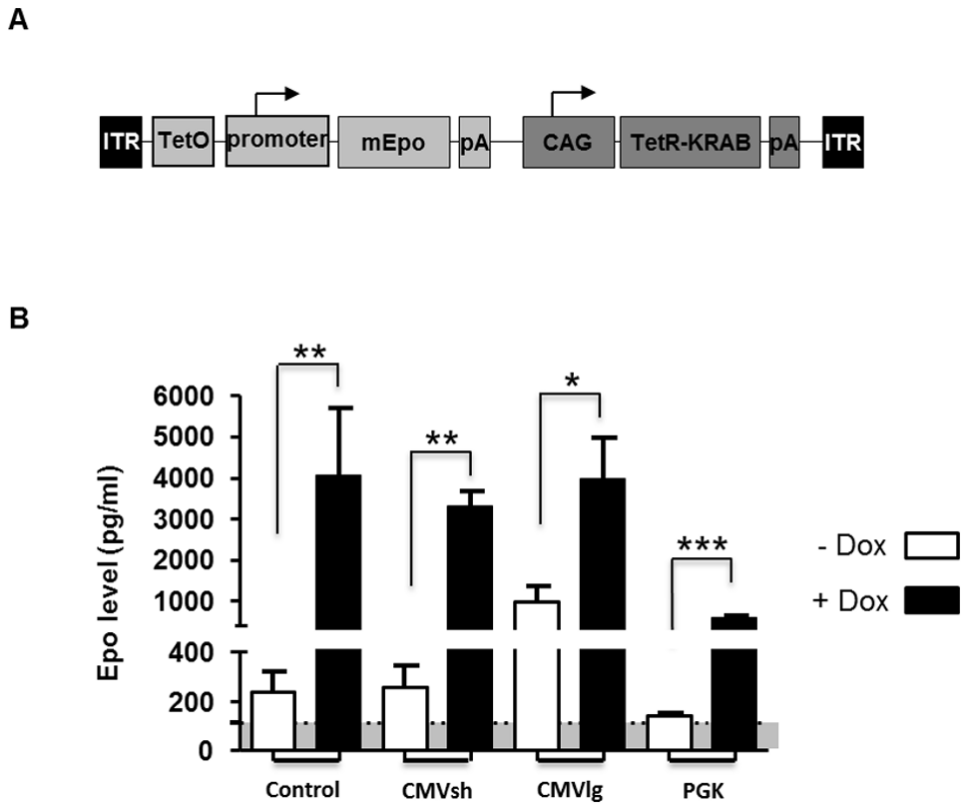
rAAV.TetR-KRAB/mEpo in the mouse muscle results in Dox-mediated transgene regulation. (**A**) Vector design: the vector encoded the murine *Epo* (*mEpo*) under the control of a *TetO*.lCMVlg, *TetO*.CMVsh, or *TetO*.PGK promoter and the trans-inhibitor TetR-KRAB under the control of the CAG promoter. pA (*mEpo*), SV40 polyadenylation signal; pA (TetR-KRAB), BGH polyadenylation signal; ITR, inverted terminal repeat of AAV2. Arrows indicate the transcription start sites. All expression cassettes were cloned between AAV2 ITRs in the same orientation (*forward*). (**B**) Follow-up of mEpo levels in animal groups before and after Dox induction. Six to 8 mice were injected IM in the *tibialis* anterior muscle with a rAAV1 vector harboring either *TetO*-CMVlg (n = 8), *TetO*-CMVsh (n = 6) or *TetO*-PGK (n = 8) promoters at a dose of 3×10^9^ total viral genomes (vg) in a volume of 30 µL. A rAAV1 vector carrying an rtTA-*mEpo* cassette (*forward* orientation) was injected at the same dose and volume in the control group (n = 8). Epo levels in the absence and in the presence of Dox are presented as the mean + SD *per* group in pg/ml. Murine Epo baseline (dotted line) was determined as the mean Epo level (115 pg/ml) in non-injected mice (n = 83). Mann-Whitney statistical test was performed between (-Dox) and (+Dox) conditions for each experimental group. ***: p<0.001, **: p<0.01, *p<0.05.

**Figure 4:**
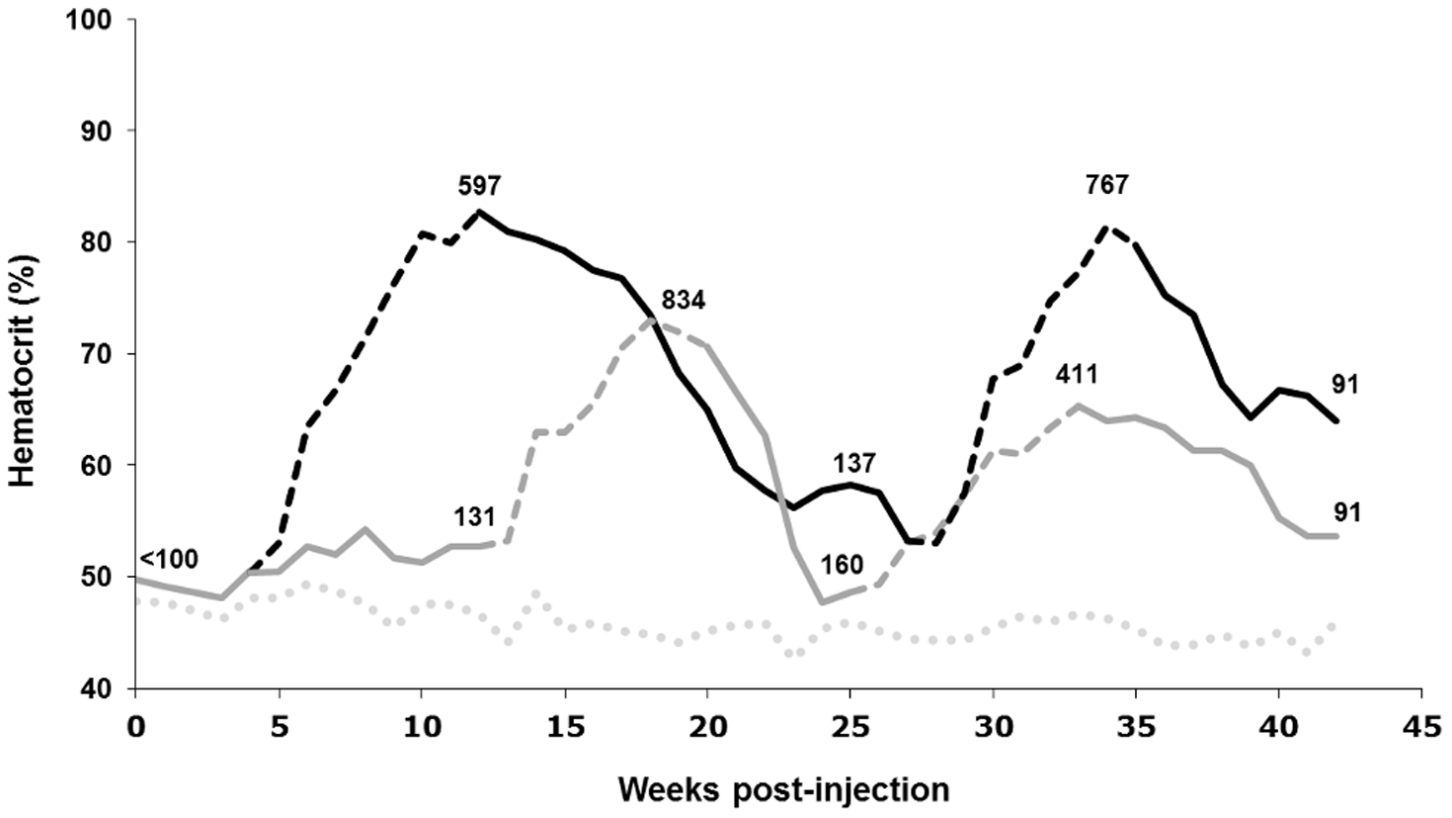
Regulation of mEpo expression using the TetR-KRAB system in mice over 42 weeks. Long-term follow up of the mouse group injected with the PGK-TetR-KRAB construct (n = 8) consisted of the monitoring of hematocrit and Epo levels under alternate Dox cycles for 42 weeks. Two subgroups of mice (n = 4 for each group, black and dark gray lines) were monitored with alternated induction kinetics. The hematocrit was measured weekly by collecting ≈40 µL of blood *via* retro-orbital puncture and is represented as mean values (%) for each group. Discontinous lines correspond to Dox administration periods, and continuous lines to periods during which Dox was withdrawn. The dotted light gray line represents hematocrit mean values in non-injected mice (n = 10 mice *per* measure). For injected mice, Epo concentration values (pg/ml) are indicated on the hematocrit graphs at the time points they were measured by ELISA, in the presence of Dox (top numbers) and its absence (bottom numbers).

For murine muscle gene transfer, our strategy consisted of constructing an AAV vector containing the TetR-KRAB driven by a CAG promoter and the reporter murine Erythropoietin (*mEpo*) gene driven by three different regulated ubiquitous promoters (**Figure 3A**): (i) the complete version of pCMV (590 pb, CMVlg) with its enhancer region and the TATA box, (ii) a shorter version of pCMV (330 pb, CMVsh) with the 3′ end of the enhancer and the TATA box, and (iii) the human phosphoglycerate kinase (PGK) promoter. The two orientations, *forward* and *opposite*, were demonstrated to be functional in rodent retinas but the *forward* construct resulted in a slightly better transgene expression regulation. Thus, we only tested in the muscle the *forward* orientation, in which both independent units have their respective promoters in the same orientation (**Figure 3A**). The three different expression cassettes were encapsidated in a serotype-1 rAAV that allows efficient gene transfer in the skeletal muscle. Previously, we demonstrated that IM delivery of a rAAV1 vector resulted in 10 times higher transduction in the skeletal muscle compared with the rAAV2 serotype in both mice and NHP models [13]. Moreover, IM administration of a rAAV1 vector has been used in patients affected with a systemic metabolic disorder, which resulted in persistent transgene expression and sustained clinical benefit [32]. Mice were injected in the *tibialis cranialis* muscle (n = 6 to 8 *per* group), and *Epo* reporter gene regulation was monitored by hematocrit values and circulating Epo levels measured by ELISA. A control group was injected with a rAAV1 vector in which the *Epo* reporter gene was under the control of the rtTA transactivator-dependent TetON system. Similar to the KRAB constructs, the two expressing units were also in the *forward* orientation, and the expression of the rtTA transactivator was driven by the CAG promoter. In the absence of Dox, the PGK TetR-KRAB group had the most repressed Epo expression; the mean of the Epo values was not higher than the basal Epo level in non-injected mice (mean of 115 pg/ml, n = 83) (**Figure 3B**). This stringent OFF state of Epo expression resulted in stable hematocrit levels in this group in the absence of induction **(Data not shown)**. In comparison to non-injected mice, a higher basal Epo expression in the absence of Dox was observed in all other groups 10 days after vector delivery. The CMVlg promoter resulted in the highest increase of hematocrit levels **(Data not shown)**, according to the highest Epo background level, in the absence of Dox (971 *versus* 115 pg/ml for non-injected mice, **Figure 3B**). During Dox induction cycles, Epo expression increased (4- to 16-fold) in response to Dox for all the tested constructs compared with non-induced mice in each injected group. Similarly to the *TetO*-CMV/rtTA control construct, when Epo expression was under the control of either the *TetO*-CMVsh or the *TetO*-CMVlg promoters in TetR-KRAB constructs, Dox induction resulted in high levels of Epo synthesis (mean of 4039, 3286 and 3953 pg/ml for rtTA, CMVsh and CMVlg promoters, respectively, **Figure 3B**). In contrast, when *Epo* gene expression was driven by the *TetO*-PGK promoter, TetR-KRAB-mediated regulation resulted in a more physiological induction of Epo expression, with a 4-fold increase compared with the non-induced mice (141 pg/ml in the absence of Dox *versus* 576 pg/ml after induction, p-value  = 0.0002, **Figure 3B**). Moreover, in this group, animals recovered background hematocrit levels more rapidly after doxycycline was withdrawn **(Data not shown)**. Thus, the combination of the *forward* orientation and the *TetO*-PGK promoter allowed successful regulation with little to no background expression in the absence of Dox. The better regulation with the PGK promoter was not due to a difference in transduction efficiency as suggested by the number of vector copies quantified in the injected muscle (**Figure S2A**). Indeed, while all the injected groups were statistically different from the mock non-injected group (P values  = 0.0007 to 0.004), no statistical difference was found between the PGK group and the other vectors (P values  = 0.14 to 0.28). We further monitored the hematocrit and Epo levels in the PGK TetR-KRAB group at regular time intervals and after several Dox cycles over a period of 42 weeks post-injection (**Figure 4**). Despite variable increases of hematocrit and Epo levels after Dox induction, responsiveness of all the mice was observed. These results indicate that the TetR-KRAB-based regulatory system was still fully functional several months after gene transfer to murine skeletal muscle, similar to the long-term regulation that was previously reported with the rtTA transactivator in mice [33].

### rAAV.TetR-KRAB/Epo in nonhuman primate muscle fails to achieve long-term Dox-mediated transgene regulation

**Figure 5:**
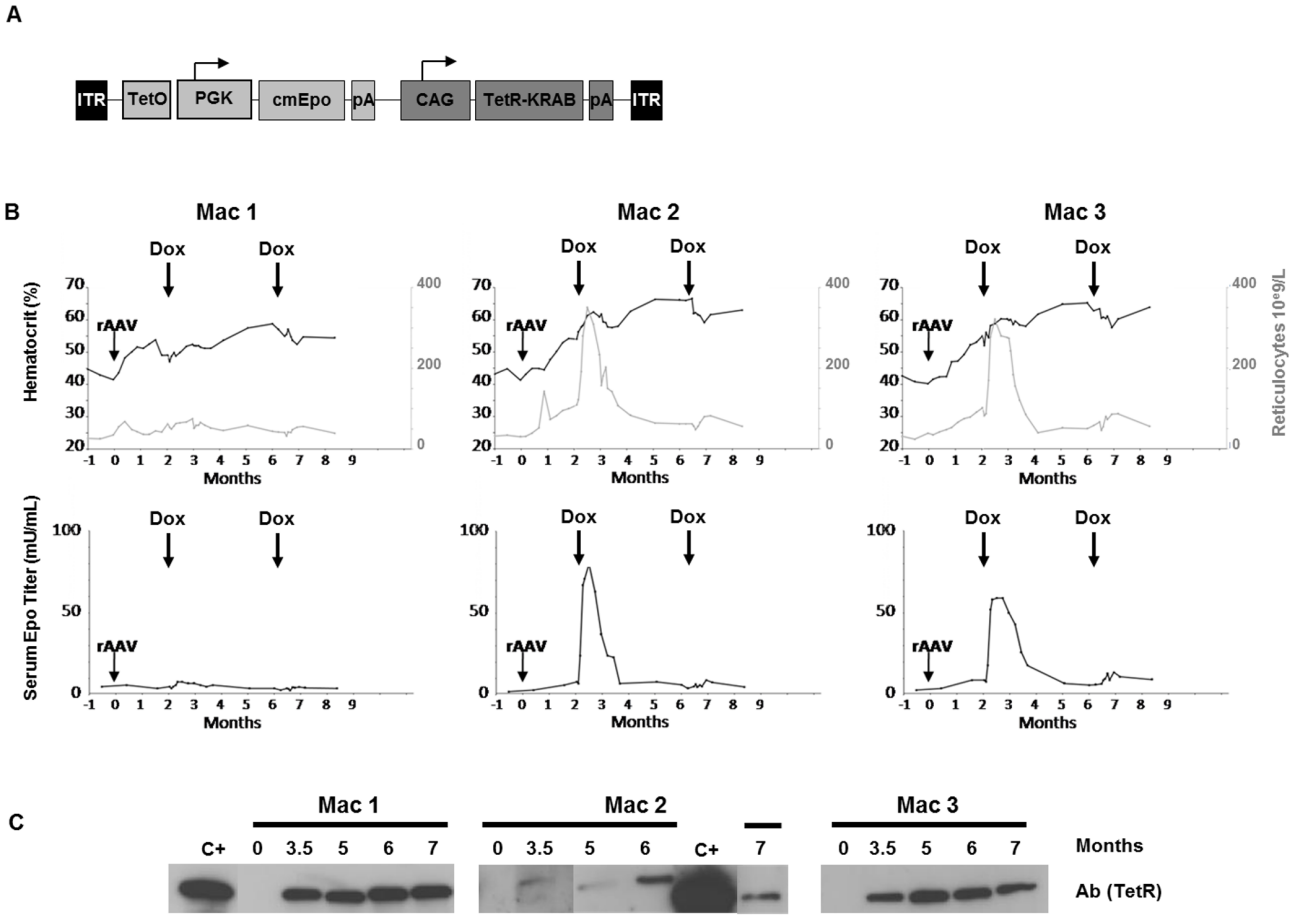
rAAV.TetR-KRAB/cmEpo in the macaque muscle results in the loss of Dox-mediated transgene regulation. (**A**) Vector design: the vector encodes the Cynomolgus macaque *Epo* (*cmEpo*) under the control of the doxycycline-inducible *TetO*-PGK promoter and the TetR-KRAB chimeric protein under the control of the CAG promoter. Arrows indicate the transcription start sites. ITR: inverted terminal repeat sequence of AAV2; pA (mEpo), SV40 polyadenylation signal; pA (TetR-KRAB), BGH polyadenylation signal. The two expression cassettes were cloned between AAV2 ITRs in the same orientation (*forward*). (**B**) Follow-up of hematocrit, reticulocyte and *serum* Epo levels in Mac 1, Mac 2 and Mac 3 injected *via* IM route with 8×10^10^ vg/kg of rAAV1-TetR-KRAB/*cmEpo* vector for over 8 months. rAAV injection and Dox administrations are indicated with arrows **Upper panel**: Hematocrit levels (%) in dark lines, and reticulocyte numbers (10^9^/L of blood) in gray lines. **Lower panel**: *Serum* Epo levels (mU/mL). (**C**) **Detection of anti-TetR IgG antibodies** (Ab (TetR)) by western-blot analysis before rAAV administration (day 0) and at 3.5, 5, 6 and 7 months post-injection. C+: positive control consisting of a commercial specific monoclonal antibody against TetR.

**Figure 6:**
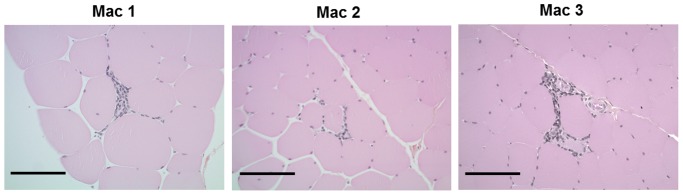
Detection of muscle lesions at the site of vector injection in macaque tibialis cranialis muscles. IM sites of rAAV injections in *tibialis cranialis* muscles were localized with tattoos performed prior to injection. Muscle samples were obtained at animal euthanasia at 2 years post-injection. HES stainings were performed on formol-fixed and paraffin-treated muscle sections. Representative pictures for Mac 1, Mac 2 and Mac 3 illustrating muscle infiltrations are shown at magnification ×20. Scale  = 100 µm.

To test the ability of the TetR-KRAB-based regulation system to establish long term transgene regulation after rAAV-based gene transfer to macaque skeletal muscle, we injected *via* the IM route a rAAV1 vector driving the expression of the homologous *Cynomolgus* macaque erythropoietin (*cmEpo*) gene under the control of the TetR-KRAB protein. The optimal construction defined in the murine studies in terms of Epo transgene expression and regulation was used: the two-expression cassettes *TetO*.PGK- cmEpo-pA and CAG-TetR-KRAB-pA were cloned in the same (*forward*) direction (**Figure 5A**). Three macaques (Mac 1, Mac 2 and Mac 3) seronegative for AAV1 serotype received 8×10^10^ vg/kg of this rAAV1 vector in the *tibialis* muscle through 5 to 8 IM injection sites. Epo secretion following Dox administration was monitored as described in our previous studies using the rtTA transactivator-based TetON system [13], [14], [19]. Upon the first Dox intravenous administration (three day Dox pulse), Epo expression was induced in two out of three macaques with Mac 1 not showing an Epo peak (**Figure 5B**). Unresponsiveness of Mac 1 was not likely due to a lower muscle transduction efficiency because comparable vector copy numbers in IM injection sites were detected in all three macaques (**Figure S2B**). Furthermore, Mac 2 and Mac 3 also had a loss of inducible Epo expression following the second Dox cycle (**Figure 5B**). In all three animals, the absence and/or the loss of Epo transgene regulation was correlated with the detection of anti-TetR IgG antibodies in the *sera*, as assessed by Western-Blot (**Figure 5C**). An ELISA assay performed at 8 months post-injection further confirmed the presence of IgG anti-TetR-KRAB antibodies, with the highest level found in the unresponsive Mac 1 (IgG titer at 1/10240 *versus* 1/1280 and 1/640 for Mac 2 and Mac 3, respectively; **data not shown**). As previously observed with the original rtTA-based regulatable system, the loss of gene regulation was associated with an immune response against the TetR-KRAB protein. The recognition of TetR-KRAB protein by the host immune system appears to be initiated earlier than we observed in our previous cohorts expressing the original rtTA transactivator [14], [19]. Indeed, IgG antibodies were detected in Mac 1 after the first Dox induction, as early as 2.5 months post-vector delivery as shown in **Figure S3A** (in contrast to a mean of 3 to 4 months post-gene transfer for the rtTA transactivator, in our previous studies), a finding that was correlated with the complete abolition of gene regulation in this animal. The humoral response against the TetR-KRAB protein was associated with muscular cell infiltrates at the sites of vector injections in all three macaques. These infiltrates were localized either in perivascular areas or surrounding some necrotic fibers and were predominantly made of mononuclear cells (**Figure 6**).

Aside from the immunotoxicity of the TetR-KRAB regulator following gene transfer in the macaque skeletal muscle, *Epo* gene regulation in this model was not as concise as in mice with the *TetO*-PGK promoter. In all NHP, Epo levels, hematocrits and reticulocyte numbers increased slightly during the 2 first months following AAV-injection and prior to the first Dox induction (**Figure 5B**). In addition and in contrast to the rtTA-based system [14], [19], Epo levels slowly decreased after Dox withdrawal, reaching baseline levels within an average of 2 months after Epo induction in Mac 2 and Mac 3. Altogether, translation of the TetR-KRAB-based platform to the NHP model not only resulted in an immune response against the regulator itself but also failed to achieve concise and sustained transgene regulation.

## Discussion

The clinical efficacy and safety of rAAV-based gene transfer will be improved with the use of systems able to turn “on” and “off” therapeutic gene expression. This is crucial for maintaining appropriate levels of gene products within the therapeutic range in order to prevent potential toxicity. It is also critical, for the ability to turn off gene expression in the case of harmful side effects. Four drug-dependent regulation systems have been described to date *in vivo*: the tetracycline (Tet) and the rapamycin regulation systems and the mammalian steroid receptor (mifepristone and tamoxifen)- and the insect steroid receptor (ecdysteroid)-based systems. Tetracycline-dependent systems have been the most widely used in rodents with sustained transgene regulation. However, when translational studies were initiated in larger animals, the development of an immune response against the rtTA transactivator was observed, leading to transient gene regulation [34]. Here, we examined *in vivo* a recent Tet-dependent system based on the TetR-KRAB regulatory protein to determine if it exhibits the features required for clinical translation, including successful assessment in large animal models. With respect to potential rAAV-based gene therapy applications targeting the skeletal muscle or the retina, we first assessed the system in rodents after either rAAV5 subretinal administration or rAAV1 IM delivery. In both gene transfer models, the major features desirable for a gene regulation system were observed: *(i)* undetectable to low reporter gene expression in the absence of Dox and *(ii)* induction of expression in the presence of Dox that is fully reversible after Dox withdrawal. Similarly, previous studies have demonstrated efficient TetR-KRAB-mediated gene regulation in rodents using either lentiviral [26] or rAAV [28], [29] vectors. For the latter viral vector system, TetR-KRAB-based regulation was demonstrated to be effective in the skeletal muscle upon IM rAAV8 administration [29] and in the liver, heart and muscle after rAAV6 intravenous delivery [28]. Another important feature for a drug-inducible system is whether gene regulation is persistent over time. In these two studies [28], [29], mice were monitored until 4 months post-rAAV injection, showing efficient transgene regulation during this time period. Here, we demonstrated in rodents the long term inducibility of the system over a longer period with a follow-up of 42 and 48 weeks post-injection after mouse muscle and rat retina gene transfer, respectively.

Because the KRAB box has demonstrated the ability to inhibit all DNA Polymerases I, II or III within a range of 3 kB of its DNA attachment site [24], we tested two expression cassettes in the retina where either the CAG promoter, driving TetR-KRAB expression, was located 2.5 kB from the *TetO* sequences (*forward* orientation) or at a distance of 4 kB in the *opposite* construction. In our gene transfer model, the *forward* orientation was as active as its counterpart, with even a slightly better gene regulation than with the *opposite* construction, as demonstrated by d2GFP quantification. This indicates that the CAG promoter was not repressed by the KRAB box in our retinal model. We further confirmed this observation in the mouse muscle, where the *forward* orientation was also able to drive *Epo* gene regulation.

In the mouse muscle, we compared three different promoters driving *Epo* gene expression to evaluate whether concise inducible gene expression is dependent on the strength of the promoter used. The long and short CMV promoter versions resulted in higher Epo basal expression in the absence of Dox, in contrast to the PGK promoter, which resulted in similar Epo levels compared with control mice. The long CMV version, with its complete enhancer region, resulted in an eight-fold Epo leakage compared with basal Epo in control mice, *versus* only 2-fold for the short CMV promoter variant. Therefore, to obtain the most concise regulation and physiological expression upon induction, promoter choice appears critical. It is desirable to develop additional strategies to restrict gene expression in the target tissue using either a specific-tissue promoter or miRNA-regulated vectors. For instance, Pichard *et al*. have recently developed a strategy combining the TetR-KRAB artificial transgene-repressor, the endogenous miRNA silencing machinery and tissue specific promoters with optimized spatial and temporal gene expression control after rAAV8-based gene transfer in mice [29].

The mechanism by which KRAB inhibits expression of genes is not entirely known. Three pathways seem to be involved: *(i)* local change in chromatin structure, *(ii)* local histone deacetylation, and *(iii)* indirect influence of the arrangement of the basal transcription machinery. Therefore, until recently, the hypothesis was that only integrated promoters could be inhibited by TetR-KRAB [24], [26]. Subsequently, KRAB/KAP1 was demonstrated to interact with and repress the episomal DNA of Kaposi's sarcoma Herpes Virus (HSHV), suppressing the virus passage from latency to lytic replication [35]. Since then, using either non-integrative lentiviral vectors [28] or rAAV [28], [29], KRAB-based repression systems have been demonstrated to be fully functional in the context of vector-derived episomal DNA. For rAAV vectors, episomal genomes exhibit chromatin-like macromolecular structures [3], and such a pseudochromatinian environment may allow TetR-KRAB to exhibit its regulatory function.

As the long-term efficiency *versus* immunogenicity of the TetR-KRAB based-system has not yet been explored in higher species, we also evaluated the system after IM delivery in mice *versus* the macaque model. Immune reactions against the chimeric transactivators emerged as a main limitation upon translation of the tetracycline- and the rapamycin-regulation systems to large animals [14], [34], [36], [37], [38]. The factors involved in this immunotoxicity are likely numerous but depend partially on the vector type and dose, the target organ and the route of vector administration, with a higher probability for immune rejection after IM vector injection [19], [36]. In this study, while long-term regulation (up to 42 weeks) was observed in mice after IM rAAV delivery, transgene regulation was only transitory in the NHP model, in accordance with other gene transfer models demonstrating IM-associated immunogenicity in higher species. The loss of gene regulation in our three macaques was correlated with the detection of antibodies against TetR-KRAB and the presence of muscle infiltrates at the site of injection, similar to what has been described for other systems. The stronger anti-TetR humoral immunity in Mac 1 is unlikely due to a higher local vector concentration in IM injection sites, which was shown to be critical for anti-transgene immunity in other models [39], [40]. In our model, all three macaques received 0.4 to 0.5×10^11^ vg *per* injection site and the number of *in situ* vector copies were comparable between Mac 1 and the rest of the cohort (**Figure S2B**). Despite their more rapid production in Mac 1, IgG anti-TetR antibodies were only detectable following the first Dox induction (performed at 2 months pi). This finding suggests that TetR-KRAB protein recognition by the host immune system could first require its Dox-mediated conformational change.

Exchanging the HSV VP16 domain of the rtTA transactivator with the human KRAB, which is over 95% homologous to the macaque Kox1 sequence, did not result in less immunotoxicity than when the rtTA transactivator was expressed from the macaque skeletal muscle. The host immune system may be recognizing the common TetR component that is of bacterial origin. Our data confirm this hypothesis, as we used the recombinant rtTA protein (and not the TetR-KRAB one that is not commercially available) for IgG antibody detection. Nevertheless, we are not able to conclude whether the KRAB component was also recognized by the macaque immune system. In our previous studies where the rtTA-protein was expressed in the macaque, we also obtained data suggesting that cellular immunity is directed against the TetR component of rtTA, not the viral VP16 protein. In an IFNγ ELISpot assay using an rtTA-derived peptide library, epitopes inducing IFNγ secretion were predominantly located within the first 110 amino-acids of rtTA, corresponding to the N-terminal of the TetR component. This was observed in 4 animals that received either a rAAV or an adenoviral vector expressing the rtTA transactivator (**Figure S4**). Regarding our macaque cohort who received the TetR-KRAB construct, we were not able to demonstrate a T cell effector response against TetR-derived peptides in an IFNγ-ELISPOT assay after PBMC and splenocyte stimulation (**Figure S3B and S3C**). This may be due to a transitory response, as T cells were harvested at the euthanasia of animals 2 years post-injection, or to the limited sensitivity of the assay. However, similar to our previous studies using the TetON system in the macaque [14], [19], mononuclear cell infiltrations were detected at the site of injection, suggesting a potential cytotoxic elimination of transduced fibers. Nevertheless, a cytotoxic-mediated loss of gene expression was not clearly demonstrated, as necrosis was only mild and muscle samples were analyzed at a late time point (2 years post-injection) when viral genomes were unexpectedly still detectable in all three macaques despite the loss of gene regulation (**Figure S2B**). Additionally, we cannot exclude that the observed infiltrations were due to another component in the vector preparation or even the viral capsid itself.

Although it is difficult to definitively incriminate TetR-KRAB rejection by the macaque host immune system in the loss of gene regulation, strategies aiming to modulate TetR-KRAB immunogenicity would appear to be desirable. One strategy to overcome the recognition of the TetR component by the macaque or human host immune systems could be the generation of new TetR variants after dominant epitope targeted mutagenesis. Nevertheless, this strategy could be limited by the existence of cryptic epitopes in the rtTA protein and the appearance of subdominant epitopes, as previously demonstrated in a HLA-A*0201 context [41]. Another strategy could be the use of a muscle specific promoter to express TetR-KRAB in order to limit antigen presenting cell (APC) transduction. Nevertheless, this strategy was not efficient at avoiding rtTA rejection in our previous macaque cohort where the trasactivator was expressed under the control of a desmin muscle-specific promoter [14], [19]. The choice of rAAV serotype is also of great importance; not only, it dictates capsid antigenicity but also modulates potential APC transduction and thus T cell responses toward encoded transgene products, such as the TetR-KRAB regulator. In this study, we used serotype 1, which has been demonstrated to result in high local vector concentrations after IM administration [42] and a higher likelihood of immunotoxicity [39]. The evaluation of other AAV serotypes could be interesting, such as serotype 8, which has already been demonstrated to be less immunogenic in the muscle [43], [44], [45].

In conclusion, our study demonstrated that gene regulation is efficient and persistent using the TetR-KRAB-based Doxycyclin-dependent system in rodents after rAAV-based gene transfer in the retina and the skeletal muscle. However, when the TetR-KRAB protein was expressed in the macaque skeletal muscle, the host immune system was triggered, similar to previous studies using other chimeric transactivators. Our findings emphasize the necessity of translational studies in larger animal models before proceeding to the clinic. Joint improvement of vector design and the development of alternative modes of delivery may improve the safety of gene-switch-based systems. Nonetheless, new developments in the field are still desirable to generate new chimeric proteins with the potential to not trigger the host immune system in large species.

## Material & Methods

### Animals and ethics statement

The study involving mice, rats and macaques was conducted between 2007 and 2010 and was approved by the internal animal care and use Committee of the Boisbonne Center (Nantes Veterinary School, ONIRIS, France) where the study was realized. This animal facility received the authorization #0056397-B delivered by the Departmental Direction of Veterinary Services (Loire Atlantique, France). All animals were handled in accordance with the Guide for the Care and Use of Laboratory Animals and in accordance with the French law concerning experimentations on vertebrate laboratory animals (Décret 87-848, 1987). C57Bl/6 6 week old male mice and adult Wistar rats were obtained from Charles River laboratory (France), and captive-bred macaques (Macaca Fascicularis) were purchased from BioPrim (Baziège, France). NHP experiments were conducted on 3–5 kg male macaques with no detectable neutralizing antibodies against AAV serotypes before inclusion in the protocol. Rats experiments were conducted under the agreement #B44–32 and mouse and NHP studies under the agreement #F44–273 delivered by the Departmental Direction of Veterinary Services (Loire-Atlantique, France). Macaque study was conducted in accordance with the recommendations of the Weatherall report: “The use of nonhuman primates in research”. The macaques were housed in an enriched environment (toys) and were under a varied feeding regimen that included fresh fruits and vegetables. In addition, they were monitored daily for health and welfare. To avoid any discomfort during and after the experiments, all procedures were carried out after animal sedation with 20 mg/kg of Medetomidine (Domitor, Pfizer, France) and 8 mg/kg of ketamine (Imalgène, Rhone Merieux, France). Intramuscular injections of rAAV vectors and intravenous injections of Doxycycline were classified as mild severity procedures. Vector administration was performed under ketamine/medetomidine-induced anesthesia maintained with 1,5% isoflurane. Primates were monitored by clinical observations, respiratory rate and temperature measurements, electrocardiography, oxymetry, and capnography. Analgesia was performed with 0,1 mg/kg morphine (Morphine, Cooper, France). During the protocol, special attention was paid to the health and welfare of animals, and blood samples were collected regularly to follow biochemical and hematological parameters, in addition to the clinical follow-up. At the end of the protocol, euthanasia was performed with intravenous injection of Sodium Pentobarbital (Dolethal, Vétoquinol, France) after 0,1 mg/kg morphine-induced analgesia.

### Vector constructs and production

rAAV1 and rAAV5 viral preparations were all manufactured using the 293 cell transfection method and purified by cesium chloride density gradients followed by extensive dialysis against phosphate-buffered saline (PBS). The viral constructs are described in **Figure 1A** for retinal gene transfer and **Figures 3A** and **5A** for muscle gene transfer in the mouse and the macaque, respectively. Briefly, TetR-KRAB was under the control of the CAG promoter, while *d2GFP* or *Epo* reporter genes were under the control of either *TetO*-CMV (either short or long versions) or *TetO*-PGK promoters. Gene accession numbers for *mEpo*, *cmEpo*, *KRAB* and *TetR* sequences are BC119271.1, M 18089, NM015394 and BAG71042.1, respectively. The two expression units were cloned either in the same (*forward*) or in the *opposite* directions between the Inverted Terminal Repeats (ITRs) of AAV2. The viral recombinant vector used in the NHP study was approved by the Scientific Committee of the "Haut Conseil des Biotechnologies" of the French Ministry of Research under reference #4597CA-II.

### Rat injections and d2GFP follow up

Wistar rats were anesthetized with an IM injection of ketamine and xylazine (50 mg/kg and 6 mg/kg, respectively). Subretinal injections were performed *via* a transscleral transchoroidal approach as previously described [46]. When needed, Dox was added to the drinking water at a dose of 10 mg/kg/day, which corresponds to a 500 µM solution, supplemented with 5% sucrose. The expression of d2GFP protein in living rats was monitored by fluorescence retinal imaging using a Canon UVI retinal camera connected to a digital imaging system (Lhedioph win software). Retinas were examined at daily intervals following injection. Identical experimental conditions and parameters were used for fluorescence fundus photography at each time point. The quantification of d2GFP in the retina was performed as described in **Materials and Methods S1**.

### Mice injections and mEpo follow-up

IM injections in mice were given in the tibialis anterior muscle after anesthesia with ketamine and xylazine. In all groups, a dose of 3×10^9^ total viral genomes (vg) of vector was injected in the muscle in a volume of 30 µL. Dox (Roxaxan P.S. 5%, Coophavet, France) was dissolved in the drinking water to a final concentration of 200 µg/mL with 5% sucrose. Blood samples were obtained by retro-orbital puncture in isoflurane-anesthetized animals. Hematocrit levels were monitored weekly and *serum* Epo levels were measured before and after Dox inductions by enzyme-linked immunosorbent assay (ELISA) using Mouse Epo Quantikine ELISA kit (R&D systems, USA). The quantification of vector copy numbers by qPCR in the muscles of mice was performed as described in **Materials and Methods S1**.

### NHP injections and cmEpo follow-up

IM injection of rAAV in NHP was conducted as described previously [42] in the absence of any immunosuppressive regimen. For blood samples, primates were anesthetized with intramuscular injection of 20 µg/kg medetomidine (Domitor, Pfizer, Paris, France) associated with 8 mg/kg of ketamine (Imalgène, Rhone Merieux, Toulouse, France). Vector administration was performed under ketamine/medetomidine/morphine-induced anesthesia maintained with 1,5% isoflurane. A total vector dose of 8×10^10^ vg/kg split over 5 to 8 pre-tattooed injection sites along the two *tibialis anterior* muscles was administered in a volume *per* site of 500 to 650 µL. The induction protocol started 2 months after rAAV1-TetR-KRAB/Epo delivery. Doxycyclin (Ratiopharm, Ulm, Germany) was given intravenously (20 mg/kg the first day, then 10 mg/kg the second and third day). Circulating Epo levels were measured by ELISA using the Human Epo Quantikine IVD kit (R&D systems, USA). The quantification of vector copy numbers by qPCR in the muscles of NHP was performed as described in **Materials and Methods S1**.

### Follow-up of anti-TetR immune responses in NHP

Detection of anti-TetR antibodies was conducted as previously described [14], [19] by Western-Blot analysis. In the absence of available recombinant TetR-KRAB protein, purified recombinant rtTA protein (Proteogenix, France) was used, only allowing the detection of antibodies directed against the TetR component of the chimeric TetR-KRAB protein. Follow-up of anti-TetR cellular immune responses in NHP was performed as described in **Materials and Methods S1**.

### Histopathological analysis

*Tibialis* muscles were sampled at animal euthanasia 2 years post-injection. Hematoxylin eosin saffron staining (HES) was performed as *per* standard histological protocols using formol-fixed and paraffin-embedded muscle sections.

## Supporting Information

Figure S1 **d2GFP fluorescence quantification in the retina after rAAV.TetR-KRAB/d2GFP administration.**
(PDF)

Figure S2 **Quantification of vector copy numbers in the muscle after rAAV.TetR-KRAB/mEpo intramuscular (IM) administration of rAAV vectors.**
(PDF)

Figure S3 **Monitoring of anti-TetR humoral and cellular immune responses in Mac 1, 2 and 3 after rAAV1-mediated TetR-KRAB expression.**
(PDF)

Figure S4 **Anti-rtTA cellular immune responses in the macaque are directed against epitopes among the TetR component and not the VP16 one.**
(PDF)

Materials and Methods S1 **Quantification of d2GFP fluorescence in the retina.** Quantification of vector copy numbers by qPCR in the muscle. Follow-up of anti-TetR cellular immune responses in NHP.
(PDF)

Checklist S1 **ARRIVES Checklist.**
(PDF)
