# Supplementary material for: Transgene Regulation Using the Tetracycline-Inducible TetR-KRAB System after AAV-Mediated Gene Transfer in Rodents and Nonhuman Primates
Source: PLoS One. 2014 Sep 23;9(9):e102538. doi: 10.1371/journal.pone.0102538 (PMC4172479; doi:10.1371/journal.pone.0102538)
Supplement: Figure S1 — d2GFP fluorescence quantification in the retina after rAAV.TetR-KRAB/d2GFP administration. (PDF) [file pone.0102538.s001.pdf]

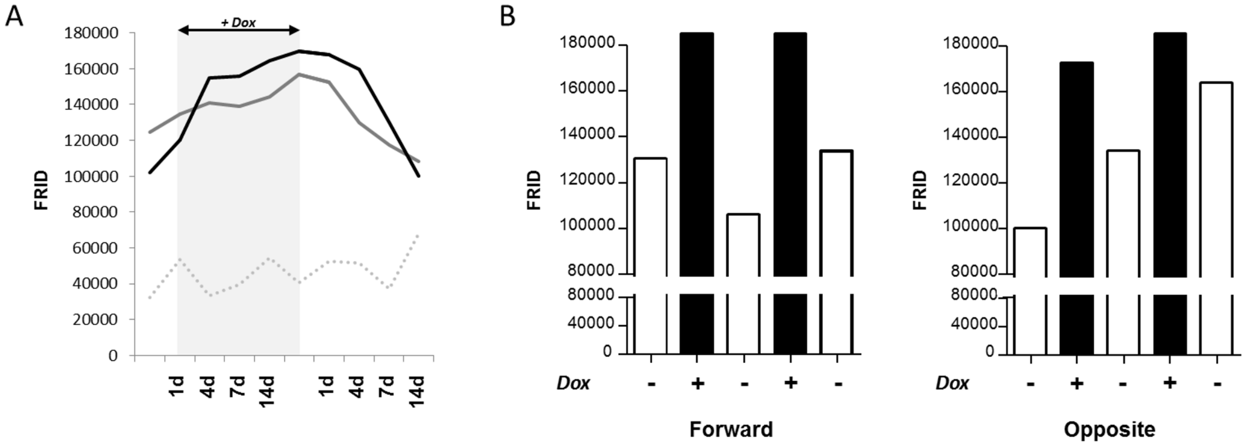

Figure S1: d2GFP fluorescence quantification in the retina after rAAV.TetR-KRAB/d2GFP administration.

(**A**) Quantification of retinal d2GFP fluorescence during induction and de-induction kinetics in representative rats subretinally injected with either rAAV2/5.d2GFP.KRAB *forward* (black line) or rAAV2/5.d2GFP.KRAB *opposite* (gray line). The corresponding eye fundi are presented in Figure 1. The expression of d2GFP was monitored and quantified after vector delivery, following Dox administration (shaded area) and Dox withdrawal during 14 days for each induction cycle at the indicated time points. **Dotted line**: quantification of the retinal background fluorescence signal in a representative mock non-injected rat. **FRID**: fluorescence raw integrated density.

(B) Long term quantification of d2GFP expression in the rat retina using the TetR-KRAB system. Expression of d2GFP was induced several times with Dox for over 48 weeks to evaluate the long-term regulation and functionality of the regulatory system after subretinal injection of rAAV2/5.d2GFP.KRAB *forward* and *opposite* vectors. The quantification is shown for one representative rat for each construct. The corresponding eye fundi are shown in Figure 2 and were taken 14 days after starting the administration of Dox **(+Dox)** or its withdrawal **(-Dox)** at 15, 20 and 38 weeks post-injection. **FRID**: fluorescence raw integrated density.
