# Supplementary material for: Transgene Regulation Using the Tetracycline-Inducible TetR-KRAB System after AAV-Mediated Gene Transfer in Rodents and Nonhuman Primates
Source: PLoS One. 2014 Sep 23;9(9):e102538. doi: 10.1371/journal.pone.0102538 (PMC4172479; doi:10.1371/journal.pone.0102538)
Supplement: Figure S2 — Quantification of vector copy numbers in the muscle after rAAV.TetR-KRAB/mEpo intramuscular (IM) administration of rAAV vectors. (PDF) [file pone.0102538.s002.pdf]

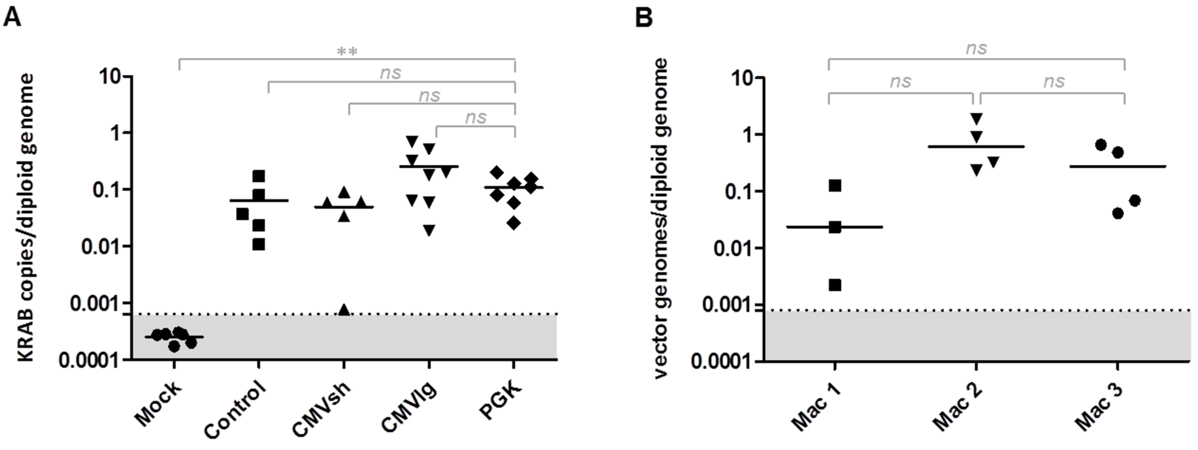

Figure S2: Quantification of vector copy numbers in the muscle after rAAV.TetR-KRAB/mEpo intramuscular (IM) administration of rAAV vectors.

(A) Viral genome copy numbers were determined by quantitative PCR in the mouse *tibialis* muscle after IM injection of a rAAV.TetR-KRAB/mEpo vector harboring either *TetO*-CMVlg, *TetO*-CMVsh, *TetO*-PGK promoters at a dose of $3x10^9$ total viral genomes (vg) in a volume of 30 µL. Muscles from a non injected group and from a group injected with a rAAV2.rtTA/mEpo cassette were analyzed as mock and control groups, respectively. Samples were obtained at animal necropsy at 10 months pi for the *TetO*-PGK group or 3 months for the other constructs.

(B) Viral genome copy numbers were determined by quantitative PCR in the macaque injected *tibialis* muscle after IM injection of rAAV.TetR-KRAB/cmEpo vector. Samples were obtained 2 years post-injection during animal necropsy Three to four intramuscular injection sites were analyzed for each macaque.

Results are expressed in (A) and (B) as viral genome (vg) *per* diploid genome (dg). The shaded area corresponds to the limit of sensitivity of the PCR, which was determined at $6.4.10^{-4}$ and 8.10^{-4} for murine and macaque qPCR, respectively. Statistical analysis was performed using a Mann-Whitney test. **: p<0.01. ns: non significant.
