# Supplementary material for: Transgene Regulation Using the Tetracycline-Inducible TetR-KRAB System after AAV-Mediated Gene Transfer in Rodents and Nonhuman Primates
Source: PLoS One. 2014 Sep 23;9(9):e102538. doi: 10.1371/journal.pone.0102538 (PMC4172479; doi:10.1371/journal.pone.0102538)
Supplement: Figure S3 — Monitoring of anti-TetR humoral and cellular immune responses in Mac 1, 2 and 3 after rAAV1-mediated TetR-KRAB expression. (PDF) [file pone.0102538.s003.pdf]

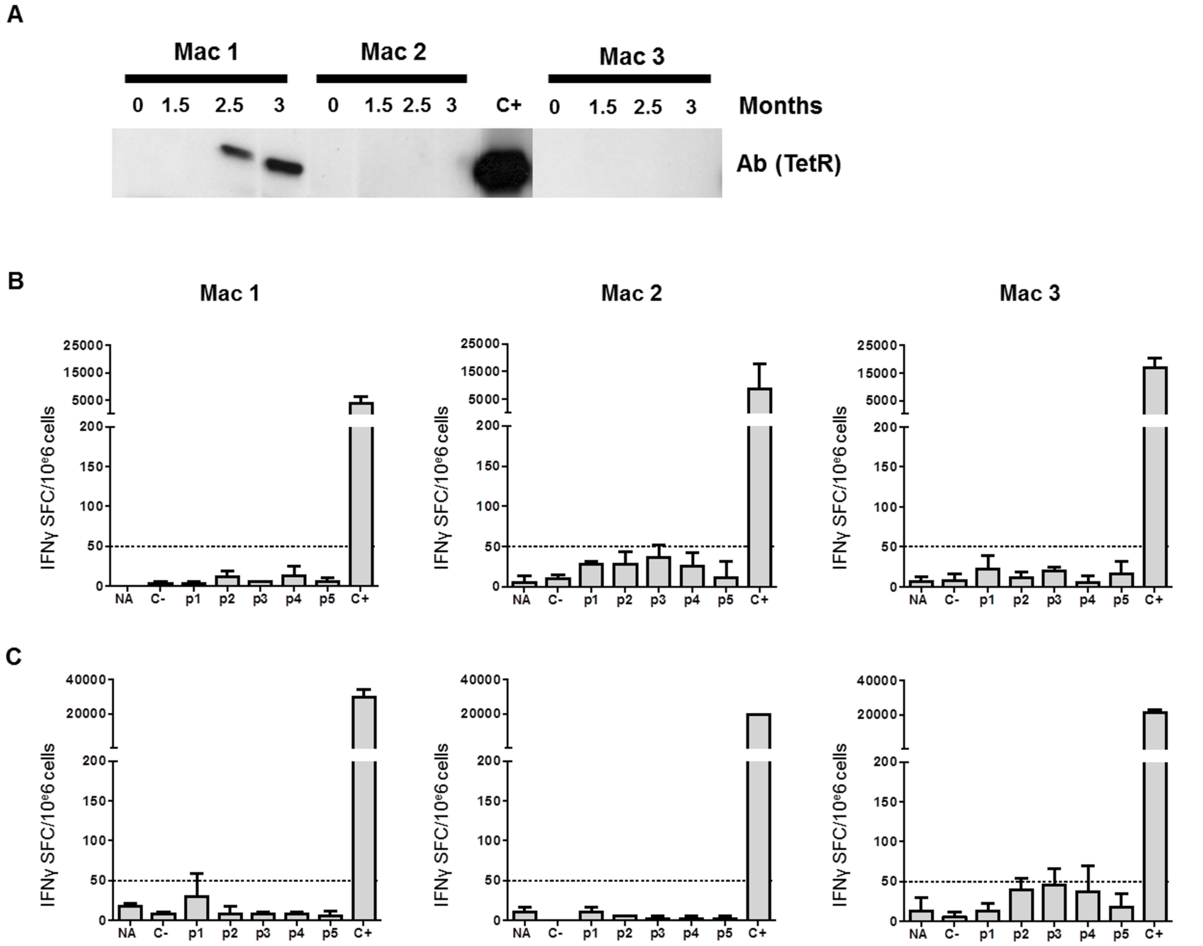

Figure S3: Monitoring of anti-TetR humoral and cellular immune responses in Mac 1, 2 and 3 after rAAV1-mediated TetR-KRAB expression.

(A) Anti-TetR humoral responses (serum IgG antibodies) were monitored by western-blot before injection (day 0) and at 1.5, 2.5 and 3 months post-injection. C+: positive control consisting of a commercial specific monoclonal antibody against TetR.

(B and C) Anti-TetR cellular responses were monitored with an IFNγ ELISpot assay using either **(B)** PBMC or **(C)** splenocytes obtained at 2 years post-injection. The cells were stimulated with an overlapping peptide library covering the rtTA sequence (15 per 10 amino acids) that was divided in 5 peptide pools (p1 to p5). (C+): Positive control consisting in PMA/ionomycin activation. (C-): Negative control consisting in an unrelated peptide pool. (NA): non-activated cells cultured in medium alone. Each condition was assessed in triplicates. IFNγ secretion was measured as Spot Forming Cells (SFC) per 10^6 cells. Threshold of positivity of IFNγ secretion (dotted line) was defined as a SFC/10^6 response > 50 SFC/10^6 cells and at least 3 times higher than the value obtained with the peptide pool negative control (C-).
