# Supplementary material for: Transgene Regulation Using the Tetracycline-Inducible TetR-KRAB System after AAV-Mediated Gene Transfer in Rodents and Nonhuman Primates
Source: PLoS One. 2014 Sep 23;9(9):e102538. doi: 10.1371/journal.pone.0102538 (PMC4172479; doi:10.1371/journal.pone.0102538)
Supplement: Figure S4 — Anti-rtTA cellular immune responses in the macaque are directed against epitopes among the TetR component and not the VP16 one. (PDF) [file pone.0102538.s004.pdf]

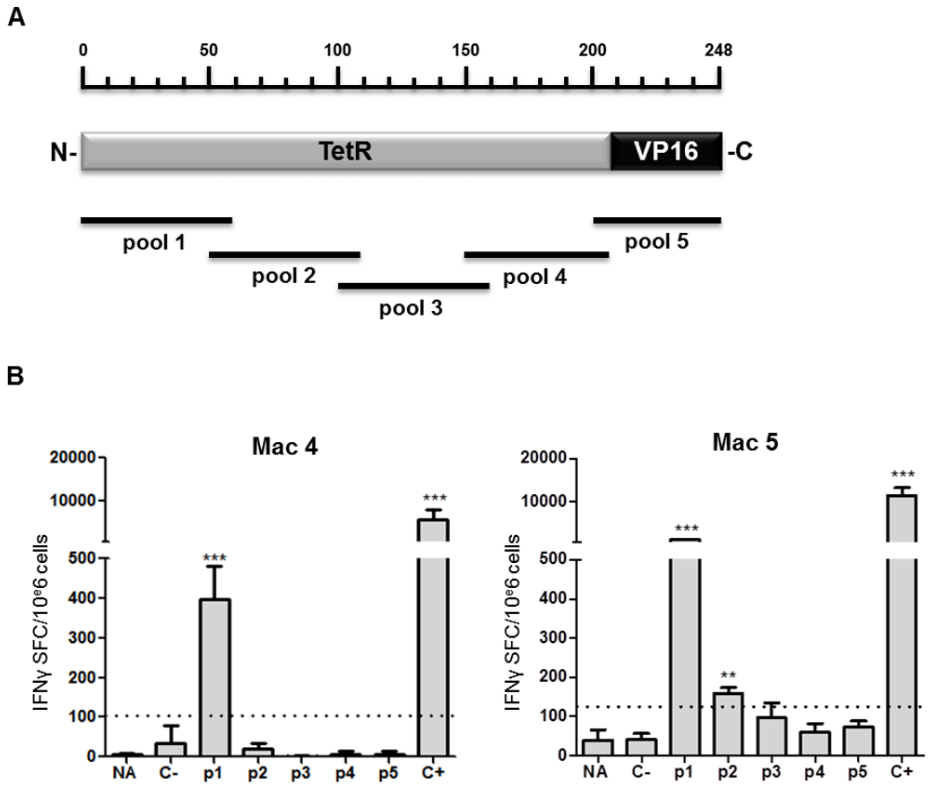

Figure S4: Anti-rtTA cellular immune responses in the macaque are directed against epitopes among the TetR component and not the VP16 one.

Anti-rtTA cellular immune response was monitored using an IFNγ ELISpot assay in 4 macaques injected in unrelated protocols with either rAAV or adenoviral vectors expressing the transactivator.

(A) The ELISpot assay was performed using an overlapping peptide library covering the rtTA sequence (15 per 10 amino acids) that was divided in 5 peptide pools. The rtTA protein sequence used to generate the peptide library was analyzed using blastp alignment tool (NCBI, USA) in order to determine the localization of each peptide pool among the protein. Pool 1: from 1 to 60; Pool 2: from 51 to 110; Pool 3: from 101 to 160; Pool 4: from 151 to 210 and Pool 5: from 201 to 248. TetR sequence is covered with pools 1 to 4 and the first peptide of pool 5. VP16-derived peptides are among pool 5.

(B) IFNγ ELISpot in 2 representative macaques (Mac 4 and Mac 5) from a group of 4 animals injected with either rAAV or adenoviral vectors expressing the transactivator rtTA in unrelated protocols. Both Mac 4 and Mac 5 presented in the figure were injected IM in a previous study with a rAAV1 vector expressing cmEpo under the control of the transactivator rtTA of the TetON system. PBMC were stimulated with rtTA overlapping peptides divided in 5 pools (p1 to p5) as described above. (C+): Positive control consisting in PMA/ionomycin activation. (C-): negative control consisting in an unrelated peptide pool. (NA): non-activated cells cultured in medium alone. Each condition was assessed in triplicates. IFNγ secretion was measured as Spot Forming Cells (SFC) per 10^6 cells. Threshold of positivity of IFNγ secretion (dotted line) was defined as a SFC/10^6 response > 50 SFC/10^6 cells and at least 3 times higher than the value obtained with the peptide pool negative control (C-). Statistical analysis was performed with the DFR(2x) test. An IFNγ response was observed against pools 1 (both Mac 4 and 5) and 2 (Mac 5) covering the 110th first amino acids of rtTA protein among the TetR component.
