# Supplementary material for: Transgene Regulation Using the Tetracycline-Inducible TetR-KRAB System after AAV-Mediated Gene Transfer in Rodents and Nonhuman Primates
Source: PLoS One. 2014 Sep 23;9(9):e102538. doi: 10.1371/journal.pone.0102538 (PMC4172479; doi:10.1371/journal.pone.0102538)
Supplement: Materials and Methods S1 — Quantification of d2GFP fluorescence in the retina. Quantification of vector copy numbers by qPCR in the muscle. Follow-up of anti-TetR cellular immune responses in NHP. (PDF) [file pone.0102538.s005.pdf]

Supplemental Materials and Methods S1:

Quantification of d2GFP fluorescence in the retina: retinas were examined at daily intervals following vector injections. Identical experimental conditions and parameters were used for fluorescence fundus photography at each time point. The quantification of d2GFP fluorescence intensity was performed using ImageJ software (NIH). The same eye fundus area was selected in each image for direct comparison. Fluorescence intensity for each area was determined as fluorescence raw integrated density (FRID).

Quantification of vector copy numbers by qPCR in the muscle: For the murine model, injected *tibialis* muscles were obtained 3 months after IM vector delivery of *TetO*-CMVlg, *TetO*-CMVsh, and rtTA-based constructs and at 10 months post-injection for the *TetO*-PGK vector. For the macaque model, 3 to 4 IM injection sites in the *tibialis* muscle were obtained 2 years post-injection during animal necropsy. Muscle genomic DNA was extracted using Gentra Puregene kit (Qiagen) according to manufacturer instructions, after tissue dissociation using Tissue Lyser II (Qiagen). Quantification of viral genomes in both murine and macaque models was performed by targeting the polyA\BGH sequence with the following primers: *forward primer* 5'-TCTAGTTGCCAGCCATCTGTTGT-3'; *reverse primer* 5'-TGGGAGTGGCACCTTCCA-3' and polyA\BGH probe 5' (6 FAM)-TCCCCCGTGCCTTCCTTGACC-3' TAMRA. Murine albumin (mALB) sequence was quantified as endogen control with the following primers: *forward primer* 5'-ACATAGCTTGCTTCAGAACGGT-3'; *reverse primer* 5'-AGTGTCTTCATCCTGCCCTAAA-3'. Macaque ε-globin gene was quantified as endogen control using the probe 5' (6 FAM)-TGCAGGCTGCCTGGCAGAAGC-3' TAMRA. qPCR was conducted using an AB StepOne Plus (Applied Biosystems). For each sample, Ct values were compared with those obtained with plasmid standard dilutions containing the polyA\BGH, the mALB or the ε-globin sequences.

Follow-up of anti-TetR cellular immune responses in NHP: Anti-TetR cellular immune responses were evaluated with an IFNγ ELISpot assay using either frozen PBMC or Splenocytes. PBMC were isolated using a ficoll gradient density separation. Splenocytes were harvested after enzymatic tissue treatment with collagenase D (Roche, 2mg/mL) and red blood cell lysis with $NH_4Cl/KHCO_3/Na_2EDTA$ buffer. The ELISpot assay was performed using an overlapping peptide library covering the rtTA sequence (15 per 10 mers, Pepscreen, Sigma), meaning that for the TetR-KRAB-injected macaques, only potential immune responses directed against epitopes in the TetR component were assessed. Briefly, the method consisted in plating $2x10^5$ cells in human anti-IFNγ (clone GZ-4, MabTech, France) pre-coated MultiScreen®HTS filter plates with polyvinyldiene difluoride membrane (PVDF, Millipore, France). Cells were restimulated with rtTA-derived peptides divided in 5 pools at a final concentration of 10µg/mL. Medium alone and an irrelevant pool of peptides served as negative controls. Cells stimulated with Phorbol- 12-myristate-13-acetate (PMA, Sigma)/Calcium Ionophore A23187 Mi Calcium Salt (Ionomycin, Sigma) served as a positive control. After incubation with biotinylated antihuman IFNγ antibody (clone 7-B6-1, MabTech, France) and ExtrAvidin® Alkalin Phosphatase (Sigma-Aldrich, France), the enzymatic reaction was revealed using NBT/BCIP (Pierce, France). Spot formation was read on an iSpot Spectrum ELISpot reader (AID, Germany), and results were expressed as the mean number of Spot Forming Cells (SFC) per $1x10^6$ cells. A positive response to any peptide pool was arbitrarily defined as a SFC/10^6 response > 50 SFC/10^6 cells and at least 3 times higher than the value obtained with the peptide pool negative control. For positive responses, statistical analysis was performed with the DFR(2x) test.
